# Mr. Simmons, in Answer to Chirurgicus

**Published:** 1805-08-01

**Authors:** W. Simmons

**Affiliations:** Manchester


### *To the Editors of the Medical and Physical Journal.*

GENTLEMEN,

In my observations on Amputation, which by your indulgence have obtained a place in the Medical Journal, I have, in common with most other chirurgical writers, assigned the invention of the ligature on the arteries for the suppression of hæmorrhage after amputation to Ambrose Paré; a merit which your Correspondent, under the signature of 'Chirurgicus,' has endeavoured to deprive him of, as appears by an article inserted in your last number, page 391. As my own communication was duly authenticated, I might have been excused from noticing any anonymous remarks upon it; but as I am less solicitous about forms, than anxious after truth, justice to the memory of Paré demands of me that the concealment of the name of the author of those remarks should be no bar to my further enquiries.

A careful review of the passages from Celsus, which 'Chirurgicus' has himself quoted, would I think convince him, that he has in one point mistaken the sense of his author, as well as the book from whence his extracts are taken, it being the fifth book of the works of Celsus,* and not the third, as 'Chirurgicus' has asserted, in which they are contained. But these are trifling mistakes compared with those which are to follow; as it will soon be made manifest, that 'Chirurgicus' has not only misapprehended Celsus, but Paré, and myself; and that his opinions on this subject are irreconcileable with the recorded facts upon it.

The very title of my own observations restricts them solely to the operation of amputation, beyond which they were never meant to be construed with any latitude; therefore to decide upon them in a general sense, is to pass sentence on grounds it was not within my intention to defend. Thus then it is certain, that the objections of 'Chirurgicus' do not apply to my own case; nor is he more happy in his reference to Celsus, for where, in this excellent writer, does he find the practice inculcated of securing the vessels by ligature after the amputation of a limb? His quotation—'Quod si illa quoque profluvio vincuntur,

 venæ,

* Lugd. Batav. 1746.

venæ, quæ sanguinem fundunt apprehendendæ, circaque id, quod ictum est, duobus locis deligandæ, intercidendæque sunt, ut et in se ipsæ coeant, et nihilominus ora præclusa habeant,' relates most distinctly to the securing of a wounded vessel by means of a *double* ligature, and has nothing to do with amputation where the second ligature could not be applied. And this was the construction put upon it by all the commentators on this particular passage, as we find it in the Varior. Auctor. in Celsum Notæ, p. 290, lib. viii. *Curatio adv. profusionem &c.* (vulneribus, quæ maxime per tela inferuntur) Casaub. & p. 291, lib. iii. *Ut et in se ipsæ coëant* (ut in se junctæ coëant. Const.

If, from the passage he had cited, 'Chirurgicus' had undertaken to prove that Celsus was acquainted with the circulation of the blood, or had ascertained in what manner hæmorrhage is suppressed by the coalescence of the sides of a wounded artery, it might have somewhat availed his purpose; but I must confess that I do not see how those who may have thought differently from him upon a point so little controverted before, can justly incur the charge of vanity, or ignorance. Charges thus loosely preferred might rob even Harvey of the discovery of the circulation of the blood; or Newton of the principle of gravitation, both of which have immortalized the names of those great men.

Not only however is Paré denied by 'Chirurgicus' the honour of the discovery for which I contend, but he is accused of having acted disingenuously, as it is said to be 'more than probable that Paré had learnt it from some Italian surgeons;' it is fit therefore that Paré should be heard in his own defence.

In lib. xii. c. 20, (Ed. 1649,) entitled, 'How to stanch the Bleeding when the Member is taken off,' he recommends to draw forth the ends of the severed vessels with the crow's beak forceps, (of which instrument a plate is given) and when they are so drawn forth, to bind them with a strong double thread.—P. 340. And in Chap. 22 of the same book, he directs us in what manner to proceed 'if any of the bound up vessels chance to get loose.' He says, 'then let the work-master take a needle some four fingers long, square, and having sharp edges, drawing after it a three or four doubled strong thread. With this let him bind the vessel in the following manner. Let him thrust his needle on the outside into the flesh, some half fingers breadth from the loosed vessel until he come to the end thereof, then let him put it about it, and bring it back again, but so that there be no more than the space of a finger's finger's breadth between the going in, and coming forth of the needle. In this space let him put a linen rag three or four times doubled, and thereupon bind somewhat strait the two ends of the thread together. For so he shall hinder the knot from hurting the flesh which lies under it in the bindings, and also add strength thereto. For so the bound up orifice of the vessel will in short space be agglutinated to the adjoining flesh, and that so firmly, that there hath never been seen any one drop of blood to have flowed from a vessel so bound up.'

Well aware of your aversion to long quotations, as rather wishing to dedicate your pages to original matter, I feel myself called upon to apologize for the length of the preceding quotation, and especially for the one that is about to follow, which, long as it is, I know not how to shorten, it is so essential to the making out of my case; and especially, as it may operate a conviction even over the scruples of 'Chirurgicus' himself. I shall transcribe the whole chapter.

" Chap. 24. What just occasion moved the author to devise this new form of remedy, to stanch the blood after the amputation of a member, and to forsake the common way used almost by all chirurgeons; which is, by application of actual cauteries?"

" Verily, I confess, I formerly have used to stanch the bleeding of members after amputation, after another manner than that I have a little before mentioned. Whereof I am ashamed, and agrieved; but what should I do? I have observed my masters, whose method I intended to follow, alwaies to do the like; who thought themselves singularly well appointed to stanch a flux of blood, when they were furnished with various store of hot irons and caustick medicines, which they would use to the dismembered part, now one, then another, as they themselves thought meet. Which thing cannot be spoken, or but thought upon without great horror, much less acted. For this kind of remedy could not but bring great and tormenting pain to the patient, seeing such fresh wounds made in the quick and sound flesh are endued with exquisite sense. Neither can any caustick be applied to nervous bodies, but that this horrid impression of the fire will be presently communicated to the inward parts, whence horrid symptoms ensue, and oft-times death itself. And verily of such as were burnt, the third part scarce ever recovered, and that with much adoe, for that combust wounds difficultly come to cicatrization; for by this burning are caused cruel pains, whence whence a feaver, convulsion, and oft-times other accidents worse than these. Adde hereunto, that when the eschar fell away, oft-times a new hæmorrhage ensued, for stanching whereof they were forced to use other caustick and burning instruments. Neither did these good men know any other course; so by this repetition there was this great losse and waste made of the fleshy and nervous substance of the part. Through which occasion the bones were laid bare, whence many were out of hope of cicatrization, being forced for the remainder of their wretched life to carry about an ulcer upon that part which was dismembered; which also took away of fitting or putting to of an artificial leg or arm instead of that which was taken off. Wherefore I must earnestly entreat all chirurgeons, that leaving this old and too too cruel way of healing, they would embrace this new, which I think was taught me by the special favour of the sacred Deity, for I learnt it not of my masters, nor of any other, neither have I at any time found it used by any. Only I have read in Galen, that there was no speedier remedy for stanching of blood, than to bind the vessels through which it flowed towards their roots, to wit, the liver and heart. This precept of Galen, of binding and sewing the veins and arteries in the new wounds, when as I thought it might be drawn to these which are made by the amputation of members, I attempted it in many, yet so that at first in my budding practice thereof, I alwayes had my cauteries and hot irons in a readinesse, that if any thing happened, otherwise than I expected in this my new work, I might fetch succour from the ancient practice, until at length confirmed by the happy experience of almost an infinite number of particulars, I bid eternally adieu to all hot irons and cauteries, which were commonly used in this work. And I think it fit that chirurgeons do the like. For antiquity, and custome in such things as are performed by art, ought not to have any sway, authority or place, contrary to reason, as they oft-times have in civil affaires; wherefore let no man say unto us, that the ancients have alwayes done thus."

And in the next chapter, (25) in recording the history of an amputation, he says, "Then presently I stanched the blood with an hot iron, for as yet I knew no other course."

I shall forbear to make any further comment on these important citations, and leave the candid and judicious reader to appreciate their respective merits, and also their bearing on the point before us.

A Plan

### A Plan for the Extermination of the Small-pox.

The cow-pox still continues to engage the attention of many of your numerous Correspondents; indeed, what subject more important can arrest it? Surely the preservation of life is the first object of our labours; and next to prevent the propagation of disease, and its consequent effects, deformity. Although the security yielded by the cow-pox from the variolous contagion is so generally admitted, I have often felt surprize, considering the magnitude of the object, that no legislative enactment has been made to at once crush the small-pox. This I think might be accomplished speedily, by ordering a general vaccination without delay; and by binding every parent to vaccinate his child within an early period after birth.

Were a general inoculation for the cow-pox to be duly enforced, all concern about the small-pox contagion would be superseded; as that dire pestilence would, by a single blow, be robbed of its victims. And by extending the same preserving principle to an early age after birth, its rage as an epidemic, on whatsoever cause it may depend, must altogether cease. Acting upon these sentiments, I have for several years made it a rule in my own practice, to inoculate within the month, where I could prevail upon my fair friends to grant my request; not only to give security to their very tender offspring, but also to prove the mildness of vaccination, and thus, by example, more quickly to circulate its advantages.

To the interposition of legislative authority on this occasion, I can see no solid objection. Laws of quarantine are deemed expedient by the legislature to prevent the inroads of the plague among us; yet, have more lives been lost by it than by the continued and destructive ravages of the small-pox?

In the numbering of the people, or the taking of the Census, I have often lamented that a clause was not inserted in the act, to compel every master to certify to the state of his family in regard to the small-pox; and in defect of security from it in any of the members, to award early vaccination. And it should have been made the especial duty of the proper officer, to ascertain that the enactments of the statute had been sufficiently complied with.

I am, &c.

W. SIMMONS.

*Manchester, May 7, 1805.*

